# The use of complementary and alternative medicine among people living with diabetes in Sydney

**DOI:** 10.1186/1472-6882-12-2

**Published:** 2012-01-12

**Authors:** Kiran Manya, Bernard Champion, Trisha Dunning

**Affiliations:** 1 Department of Medicine, University of Sydney, Nepean Hospital, Derby Street, Kingswood, 2751, Sydney, NSW, Australia; 2 Department of Nursing and Midwifery, Deakin University, Barwon Health, 229 Ryrie Street, Geelong, 3220, Victoria, Australia

## Abstract

**Background:**

Complementary and alternative medicine (CAM) is common in patients with chronic disease such as diabetes mellitus. The primary objective of the study was to determine the overall prevalence and type of CAM use in individuals with diabetes mellitus (DM) in Western Sydney and to compare the prevalence and factors associated with CAM use with the literature.

**Methods:**

A multicenter cross-sectional study was undertaken using a self-completed questionnaire distributed to patients with DM attending a public hospital and specialist endocrinology clinics in the region. The type of DM and pattern of CAM utilisation were analyzed.

**Results:**

Sixty nine people responded to the questionnaire: age range of 18-75 years during a twelve week collection period. Overall, 32 respondents with diabetes were using some form of CAM, resulting in a utilisation rate of 46.3%. Twenty of the 32 CAM users used CAM specifically to treat their diabetes accounting for 28.9% of the respondent sample population. Multivitamins (40%), cinnamon, Co-enzyme q10 and prayer were the most frequently used CAM modalities. There was no significant difference between males and females, age range, income or diabetes complications between CAM and non-CAM users. (*p *values each > 0.05) The factor most significantly associated with CAM usage was being born overseas (*p *= 0.044).

**Conclusions:**

Almost half the respondents (46.3%) used CAM: 28% used CAM specifically to treat their diabetes. Individuals born overseas were significantly more likely to use CAM than those born in Australia. Other factors such as age, gender, wealth and duration of living with diabetes were not associated with higher rate of CAM usage.

## Background

Diabetes Mellitus (DM) is recognized as a major international health problem leading to significant morbidity and mortality and is the fastest growing chronic disease in Australia. In fact, DM has been identified as Australia's fifth national health priority since 1996.

The baseline 1999/2000 AusDiab study showed 7.5% of the Australian population aged 25years and older had diabetes, including 8.0% of males and 7.0% of females [1]. In people 75yrs and over 23.6% had diabetes, most had type 2 diabetes(T2DM) but the estimates include type 1(T1DM).

Complementary and alternative medicine (CAM) is defined as a group of diverse medical and health practices that are not presently considered to be part of conventional medicine.

The conventional pharmacological treatment of Type 1 diabetes (T1DM) is insulin. The different therapies available for Type 2 diabetes (T2DM) include lifestyle modification of diet and exercise, oral medications and insulin. One of the most commonly used conventional medicines for T2DM is metformin, which was originally derived from *Galega officinalis *(French lilac or goat's rue), once considered a complementary medicine (CAM) used to treat diabetes [2].

Approximately half the Australian population uses CAM and spent an estimated $2.3 billion on CAM in 2000, which is nearly four times the public contribution to all pharmaceuticals [3]. The utilization of complementary health practitioners has also increased in recent times. An Australian National Health Survey in 2004-05 showed that 3.8% of the population (~748,000 people) had seen one of seven selected complementary health practitioners compared to 2.8% of the population in 1995. The most commonly consulted therapists were chiropractors, naturopaths and acupuncturists [4]. The actual prevalence of Australian CAM usage can be based on South Australian data where CAM use is reported to range between 48 and 53% [5].

Whilst there is little published literature on the prevalence of CAM usage in the Western Sydney region, a 1996 study reported 52% of 325 people and their carers attending an inner metropolitan Sydney teaching hospital Emergency Room used CAM, which is consistent with existing Australian and overseas data [6].

When considering the use of CAM amongst people living with diabetes, a North American study has shown the prevalence to be between 31% and 57% [7]. The current literature suggests female gender, greater wealth, higher educational status and having a chronic disease are all factors significantly associated with CAM use [8]. In addition, diabetes is an independent predictor of CAM use in the general population [9]. Most people with diabetes use conventional medicines and there are risks of interactions and adverse effect as well as unknown benefits associated with concurrent CAM- usage.

The primary aim of the research was to identify the prevalence and type of CAM usage by people with diabetes in Western Sydney. The secondary aim was to identify the demographic, socioeconomic and disease-specific features associated with CAM use and compare these factors to current literature.

## Methods

A cross sectional survey was distributed over twelve weeks between June and August 2008. A two stage sampling design was conducted which consisted of a pilot test followed by a cross-sectional multicenter study using self-completed anonymous questionnaires. The questionnaire was designed by the researchers following a literature review to identify the type of CAM previously used by people living with diabetes. The Sydney West Area Health Service (SWAHS) Scientific Advisory and the Human Research Ethics Committee (HREC), Nepean Campus approved the project (HREC Reference Number: 08/027/08/NEPEAN/42).

The pilot test involved the researcher distributing the questionnaire to five people with diabetes at a tertiary teaching hospital to establish face validity and identify any questions that needed to be altered prior to using the questionnaire in the main study. The pilot test was important to ensure the target audience understood the questions; the wording was appropriate and would yield the required data. Content validity was established by one researcher with expertise in CAM use and questionnaire design through a comprehensive literature review of similar studies and surveys. The pilot study was successful and no questions were modified prior to the main study.

The questionnaire comprised of 32 questions using a combination of open and closed question response formats [See Additional File 1]. Questions were divided into three domains related to demographic data, diabetes-related information and CAM usage. Socioeconomic information sought included gender, age group, educational, marital and employment status and country of birth. Diabetes-specific information included type and duration of diabetes, frequency of blood glucose monitoring, last glycosylated haemoglobin A1c level, and presence of any diabetes complications. CAM-related information included type of CAM used and the reason for use.

Following a literature review, the researchers grouped the CAM into three broad categories to account for the potential variety of therapies used, which included: vitamins and minerals, herbal medicines and relaxation and other therapies. Information was collected about the following CAM therapies:

Vitamins/minerals: chromium, vitamin E, Co-enzyme Q10, L-carnitine, selenium, vitamin C, Vanadium, and magnesium.

Herbal products: ginseng (*Panax quinquefolium*), garlic (*Allium sativum*), onion (*Allium cepa*), ivy gourd (*Coccinia grandis*), holy basil (*Ocimum tenuiflorum*), cinnamon (*Cinnamomum verrum)*, fenugreek (*Trigonella foenum-graecum*), milk thistle (*Silybum mariunum*), bitter melon *(Momordica charantia) *and Gymnena sylvestre.

Relaxation and other therapies: acupuncture, tai chi, massage, yoga, prayer, essential oils, and reflexology.

Although botanical names are cited here, the common names were used in the questionnaire because most respondents were more likely to recognise them.

The inclusion criteria included

• Those aged 18 yrs and over

• Known T1DM or T2DM and able to give informed consent to participate.

The exclusion criteria included

• Inability to communicate in spoken or written English

• Age under 18

• Those having participated in the pilot test.

• Those with intellectual disability or active psychiatric disease that prevented them from giving informed consent.

Following ethics approval, the main study involved distributing the questionnaire to individuals with diabetes in two separate private endocrine clinics in Parramatta and public outpatient clinics and medical wards of the Nepean Hospital in Western Sydney over a 12 week period. These centers were selected because the researcher was able to access the relevant clinics to recruit participants.

The researchers estimated that there were approximately 8 patients per week in both of the endocrine clinics and overall 24 patients from the public outpatient clinics and wards of Nepean hospital during the 12 week collection period, thus a total estimated sampling population of 120 individuals in Western Sydney. From this estimated sample, there were a total of 69 respondents.

In each setting, the treating endocrinologist or clinic health staff asked people with DM consecutively whether they would be willing to complete the anonymous questionnaire. A participant information statement that explained the aims of the project and indicated that the return of the questionnaire would be regarded as consent to use the anonymous information for the research purposes outlined was attached to each questionnaire.

### Data analysis

Qualitative and quantitative data analysis processes were used to analyse the data. The data analysis was conducted using Statistical Package for the Social Sciences (SPSS; SPSS Inc, Chicago, IL) software 9 version 12) for Windows. Results for CAM and non-CAM users were recorded as mean ± standard error and, where appropriate, analysed using standard non-parametric methods including Chi-squared test and Fisher Exact analysis. A *p *value of 0.05 was used to determine any statistically significant differences between CAM and non-CAM users. The qualitative content analysis of open-ended questions was undertaken using the framework method [10]. The framework method consists of a five-step process that involves becoming familiar with the data, identifying a thematic framework, indexing and charting key themes and mapping and interpreting the findings.

## Results

Overall, 69 questionnaires were completed during the twelve week collection period from an estimated sample population of 120. Of the 69 respondents, 32 used CAM for general health and 20 of these used CAM specifically to treat their diabetes. Table 1 shows the proportions of CAM usage based on demographic and diabetes specific related data.

**Table 1:** Proportion of CAM use related to demographic and diabetic related data and the statistical significance of these factors

Demographic Factors			
**Category**	**Using CAM**	**No CAM Use**	**P Value**

*Male*	17/*35 *(49%)	18/*35 *(51%)	
*Female *	15/*34 *(43%)	19/*34 *(57%)	0.867

*Australian born*	17/*45*(38%)	28/*45 *(62%)	
*Overseas born *	15/*24*(63%)	9/*24 *(37%)	0.044*

*Working*	15/*29*(52%)	14/*29*(48%)	
*Not Working *	17/*40 *(43%)	23/*40*(57%)	0.419

*Income < $1000/wk*	21/*44 *(47%)	23/*44*(53%)	
*Income > 1000/wk*	11/*20 *(55%)	9/*20 *(45%)	0.948

*Tertiary Qualifications*	11/*20 *(55%)	9/*20 *(45%)	
*Other Qualifications*	21/*44*(48%)	24/*44 *(52%)	0.633

*Married*	15/*35*(43%)	20/*35*(57%)	
*Not Married *	17/*33*(51%)	16/*33*(49%)	0.832

**Diabetes-specific Data**			

**Category**	**Using CAM**	**No CAM Use**	**P Value**

*Type 2 DM*	24/*50*(48%)	26/*50*(52%)	0.704
*Type 1 DM*	5/*14*(36%)	9/*14 *(64%)	

*> 10 yrs Duration*	14/*30 *(47%)	16/*30 *(53%)	0.603
*< 10 yrs duration*	17/*32 *(53%)	15/*32 *(47%)	

*Complications*	17/*31 *(55%)	14/*31 *(45%)	0.325
*No Complications*	14/*33 *(42%)	19/*33*(58%)	

*Oral Meds/insulin*	30/*64*(47%)	34/*64*(53%)	0.408
*Diet/exercise only*	1/*4 *(25%)	3/*4 *(75%)	

*HbA1C < 10*	23/*45*(51%)	22/*45 *(49%)	0.180
*HbA1c > 10*	9/*21*(43%)	12/*21*(57%)	

*Statistical Significance of Difference P ≤ 0.05

Thirty two respondents (46.3%) used one or more CAM therapies either for diabetes or general health purposes. Of these, 12 (37.5%) used CAM for both general health and diabetes and 20 (62.5%) used CAM specifically for their treatment of diabetes.

Pearson Chi square was used to determine associations among demographic and diabetes specific data in relation to CAM use. Some of the demographic factors of interest included gender, weekly income, educational status, country of birth, working and marital status. Significantly, overseas born individuals with diabetes were more likely to use CAM, 15/24 (63%) compared with 17/45 (38%) Australian born respondents (p = 0.044).

The questionnaire asked respondents to list their macro vascular or micro vascular complications. In total, 17 of the 31 (55%) patients who used CAM for any purpose had one or more diabetes complications but there was no significant association between the presence of diabetic complications and the rate of CAM use. There was no statistically significant data between the type of diabetes, duration or most recent HbA1C and the use of CAM.

Of the 20 respondents who used CAM specifically for the treatment of diabetes, the most common types used included multivitamins(40%) and cinnamon(25%), prayer(25%) and coenzyme q10(25%)

The final question asked respondents to indicate whether or not they would be willing to use CAM in the future, regardless of their current CAM usage. Sixty one responded. Of these, 41 (67%) stated they would be willing to use CAM, nine indicated they would not use CAM and 11 were unsure.

The different types of CAM used to manage diabetes are shown in table 2. Whilst the majority of respondents used CAM for diabetes, only one respondent suggested he or she used CAM to control their blood glucose levels.

**Table 2:** Types of CAM therapies used specifically to treat diabetes

Type of CAM used for DM	Number of CAM users (n = 20)
Multivitamin	8/20

Cinnamon	5/20

Co-enzyme Q10	5/20

Prayer	5/20

*Garlic*	4/20

Magnesium	3/20

Vitamin E	3/20

Massage	2/20

Fish oil/Omega 3	2/20

Yoga	2/20

Vitamin D	2/20

*Gymnena slyveste*	1/20

Vitamin B	1/20

Iron	1/20

Fenugreek	1/20

American Ginseng	1/20

Relaxation therapy	1/20

Jerusalem Artichokes(Helianthus tuberosus)	1/20

## Discussion

The overall prevalence of CAM use in Western Sydney (46%) was similar to previously published prevalence data in South Australia (~48-53%) and inner metropolitan Sydney (~52%). In comparison to overseas literature, the prevalence of CAM use in people with diabetes in Western Sydney was consistent with the UK (46%) [11] and lower than similar studies in the USA (73%) [7], India (68%) [12], Mexico (62%) [13] but higher than in Saudi Arabia (30%)[14].

The majority of patients in the study were selected either in hospital or private endocrine clinics. It is possible that they had a longer duration of disease, poorer control or higher rates of complications leading to hospitalization. Thus, some of these factors may have influenced the overall prevalence of CAM use and need to be considered when compared to the general Australian diabetic population.

The availability of CAM in the Western World (USA, UK) is similar to Australia. It is predominantly privately funded and freely available from pharmacies or health food stores. However, there may be differences in types of CAM available and cultural acceptance between Australia and countries like India or Mexico which may explain the lower prevalence rate in our study.

In contrast to the literature, this study did not show any association between CAM usage and females who are wealthy and highly educated. The main reason these factors were not proven significant was due to small sample size which may not be representative of larger population based studies. In addition, there are a higher proportion of people from lower socioeconomic status and levels of educational attainment that live in Western Sydney.

There were no statistically significant relationships between CAM usage and duration of diabetes, type of treatment and glycosylated HbA1c levels. We cannot assume that the presence of complications leads to higher rates of CAM usage. There is scope for prospective studies of CAM use amongst people newly diagnosed with diabetes and throughout the progression of their disease. This would assist in delineating whether the development of complications leads to higher rates of CAM usage. Our study did not investigate whether the presence of other co-morbid chronic illness affects the rate of CAM usage. Thereby, it was not possible to know whether the presence of diabetes alone was an independent predictor of CAM usage.

This study has shown that being born overseas is associated with a higher rate of CAM usage. This could be due to experience, upbringing, and belief system or in acceptance of CAM therapies. Approximately one third of the Western Sydney population were born overseas and migrated to Australia, significantly more than other regions. Thus, it may be difficult to extrapolate this data to the general Australian diabetic population. This association also warrants further investigation in Australia and in other countries.

The majority of respondents (67%) indicated they would be willing to use CAM for diabetes in the future if they had positive information about the benefits from their health care providers. This would suggest that individuals living with diabetes feel CAM may be a safe option and open to compliance with these therapies.

The most common types of CAM used were multivitamins, cinnamon, prayer and coenzyme q10. Of these treatments, cinnamon has had conflicting data on efficacy in the reduction of serum glucose levels. It is apparent that some of the survey respondents are aware of a potential benefit and are using this medication as an adjunct treatment for their diabetes. However, further clinical trials into the effect of cinnamon in addition to the use of conventional pharmacological treatment are warranted [15,16].

The study showed that prayer remains a common form of CAM used by those with diabetes which is consistent with a previous study [17]. This suggests that individuals with chronic disease often believe in spiritual practices that aid in their personal healing process.

Whilst the study indicated significant use of multivitamins and co-enzyme q10, single herbal remedies such as *Gymnena slyveste*, American ginseng and Jerusalem Artichokes (*Helianthus tuberosus*) was low. One respondent commented that Jerusalem artichoke reduced his or her blood glucose levels although specific details were not given.

The major limitation of the study was its small sample size. Some of the respondents choose not to answer certain questions related to income, marital status and tertiary qualifications. The sampling population of 120 was an estimate based on the number of patients who attended the two endocrine outpatient clinics and Nepean Hospital and were available to complete the questionnaire during the twelve week collection period. The exact rate of refusal to complete the questionnaire was not recorded; therefore, an exact response rate could not be calculated.

## Conclusion

Respondents with diabetes who participated in the study frequently use CAM to treat their diabetes and for general health. It appears that the majority of patients with DM who use CAM do so for the treatment of their diabetes. Health care professionals should be aware that people with diabetes use CAM and take a thorough history to document any such therapies and monitor outcomes to note benefits or potential side effects.

## Competing interests

The authors declare that they have no competing interests.

## Authors' contributions

KM was involved in study design, literature review, data collection, statistical analysis and the main author writing the paper. BC was involved in project implementation and supervision. TD provided advice on questionnaire development and involved in reviewing the paper for submission. All authors have read and approved the final manuscript.

## Pre-publication history

The pre-publication history for this paper can be accessed here:

http://www.biomedcentral.com/1472-6882/12/2/prepub

## Supplementary Material

Additional file 1 **Questionnaire on the Use of Complementary and Alternative Medicine among People living with Diabetes in Sydney**. The distributed questionnaire collecting demographic, diabetes specific and CAM related data. Click here for file
