# Supplementary material for: The use of complementary and alternative medicine among people living with diabetes in Sydney
Source: BMC Complement Altern Med. 2012 Jan 12;12:2. doi: 10.1186/1472-6882-12-2 (PMC3295731; doi:10.1186/1472-6882-12-2)
Supplement: Additional file 1 — Questionnaire on the Use of Complementary and Alternative Medicine among People living with Diabetes in Sydney. The distributed questionnaire collecting demographic, diabetes specific and CAM related data. [file 1472-6882-12-2-S1.DOCX]

Additional File 1:

Title: Questionnaire on the Use of Complementary and Alternative Medicine among People living with Diabetes in Sydney.

Description: The distributed questionnaire collecting demographic, diabetes specific and CAM related data.

**Demographics**

1. Have you been asked to complete this survey before? If yes, please return the questionnaire to staff. If no, please continue.
2. Are you male or female? **Please place a cross in the box.**

Male Female

1. Were you born in Australia? **Please place a cross in the box.**

Yes No

If you were born outside Australia, in which country were you born?


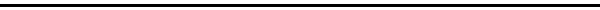


1. Please say how old you are? **Please place a cross in the box.**

18-35 36-50 51-65 >65

1. What religion are you?


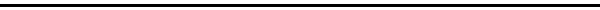


1. Are you currently: **Please place a cross in the box.**

Working Studying Retired Not working

1. What is your total gross household income per week? **Please place a cross in the box**

less than $500 a week $500 to $1000 a week more than $1000 a week

1. What is your highest level of education? ( High School, Trade, Course Diploma or University Degree)


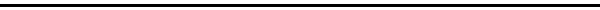


1. What is your marital status?


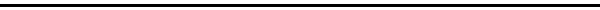


**Diabetes**

1. What type of diabetes do you have? **Please place a cross in the box**

Type 1 Type 2 Not sure

1. How long have you had diabetes? **Please place a cross in the box**.

Less than 5yrs Between 5 - 10 yrs

Between 11-20 yrs More than 20 yrs

1. What is your height (centimeters or feet/inches) and weight (kilograms)?

Height: Weight:


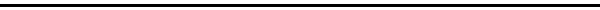


*Uncontrolled diabetes can cause long term complications due to damage caused by high blood glucose to the small and large vessels in the body.*

1. Do you have any of the following long term complications of diabetes?

**Please place a cross in the box**

Peripheral Neuropathy (pins and needles or loss of feeling in the hands or feet)

Kidney disease

Eye disease

Heart disease

Stroke

Peripheral Vascular Disease (Pain in your legs when you walk short distances)

1. What treatment do you take for your diabetes? **Please place a cross in the box**

Just diet and exercise

Oral medications

Oral medications and insulin

Insulin only

1. Do you regularly check your blood glucose levels by fingerprick? **Please place a cross in the box**.

Yes No

If yes, please tick how many times per day you do a test.

Once Two to Four More than Four

1. Do you know what your last 3 month blood glucose level (HbA1c) was? **Please place a cross in the box**.

< 7 mmol/L 7.1 - 10 mmol/L 10.1-12 mmol/L > 12mmol/L

**Complementary and Alternative Medicine (CAM)**

CAM is a group of diverse treatments that are not currently considered to be part of conventional medicine. Some commonly used CAM include vitamins/minerals or herbal products or different types of relaxation therapies.

**Complementary medicine is used together with conventional medicine**.

**Alternative medicine is used in place of conventional medicine.**

Do you use any of the following as complementary or alternative medicine to specifically help manage your diabetes? **Please place a cross in the box beside each type of CAM you are *currently* using.**

**Vitamins or Minerals**

Chromium Vitamin E

Co-enzyme Q10 Magnesium

L-Carnitine Vanadium

Selenium Vitamin C

**Please list any other vitamins or minerals you are using to manage your diabetes**


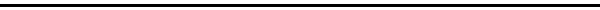

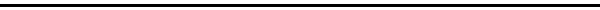


**Herbal Medicines**

American Ginseng Gurmar

(*Gymnena sylvestre*)

Ivy Gourd (*Coccinia indica*) Garlic

Onion Fenugreek

Holy Basil Milk Thistle

Cinnamon Balsampear

Prickly pear Mushrooms

**Please list any other herbal medicine you are using to manage your diabetes**


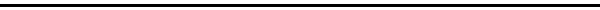


Other CAM

Acupuncture Prayer

Aromatherapy Reflexology

Essential Oils Relaxation therapy

Massage Yoga

Please list any other CAM you are using to help manage your diabetes


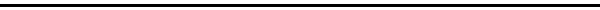


1. Have you used any of these CAM in the past to help manage your diabetes?

If yes, please list the CAM you used and why you stopped using them in the table below:

| Name of CAM | Reason it was stopped |
| --- | --- |
|  |  |
|  |  |
|  |  |
|  |  |

1. Do you use CAM for any other medical condition or for your general health (other than your diabetes)? **Please place a cross box.**

Yes No

If yes, what is it and why do you use it?


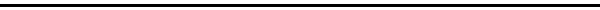

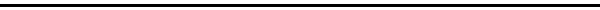

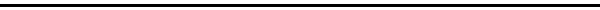


19) Who recommended CAM to you? **Please place a cross in the box.**

Naturopath Chiropractor Podiatrist

Doctor Nurse Friend

Family member Internet

Advertisement (TV/radio or magazine)

Other , please state


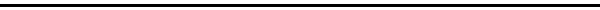


1. Where do you get your CAM from? **Please place a cross in the box**

Pharmacy Health Food Store

Doctor Alternative Health Practitioner Other

If other or from an alternative health pracitioner, please specify:


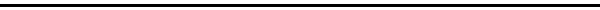


1. How much money do you spend on CAM per month? **Please place a cross in the box**.

Less than $100 Between $100-$400 More than $400

1. What is the main reason you use CAM?


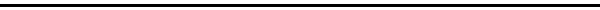

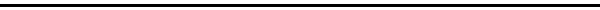

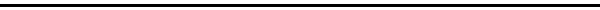


1. Have you had any side effects from the use of CAM? **Please place a cross in the box**.

Yes No Don’t know

If Yes, Please state what happened. What do you think caused it?


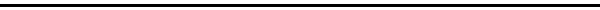

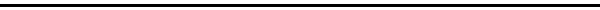

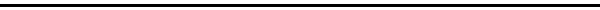


1. Do you have a regular General Practitioner who helps to manage your diabetes? **Please place a cross in the box**

Yes No

1. Do you have a regular Diabetes Specialist who helps to manage your diabetes? **Please place a cross in the box**

Yes No

1. If you are currently using any type of CAM for any reason , does your GP know you are using it? **Please place a cross in the box**

Yes No Not Sure

1. If you are currently using any type of CAM for any reason, does your Diabetes Specialist know about your use of it? **Please place a cross in the box**

Yes No Not Sure

1. Have you ever discussed using any type of CAM therapies with your General Practitioner? **Please place a cross in the box**

Yes No

1. Have you discussed using CAM therapies with your Diabetes Specialist? **Please place a cross in the box**

Yes No

1. Have you visited any General Practitioner or Diabetes Specialist in the last 3 months? **Please place a cross in the box**

Yes , for my diabetes Yes, but not for my diabetes No

1. Have you visited any alternative health practitioner in the last 3 months? **Please place a cross in the box**.

Yes , for my diabetes Yes, but not for my diabetes No

If Yes, Please specify what sort of practitioner and the reason you saw them.


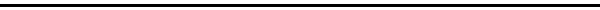

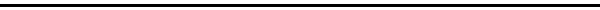

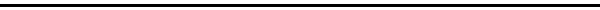


1. If you do not currently use any CAM, would you consider using it to help treat your diabetes in the future if you had positive information about its benefits from your health care provider? **Please place a cross in the box.**

Yes NO Not Sure

Please give reasons for your answer.


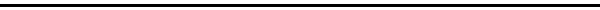

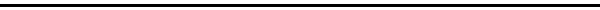


**Are there any other comments you would like to make -**


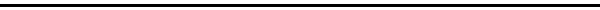

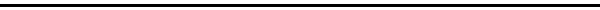


Thank you for your time completing this questionnaire
